# Common Mental Health Disorders among Informal Waste Pickers in Johannesburg, South Africa 2018—A Cross-Sectional Study

**DOI:** 10.3390/ijerph16142618

**Published:** 2019-07-23

**Authors:** Matimba Makhubele, Khuliso Ravhuhali, Lazarus Kuonza, Angela Mathee, Spo Kgalamono, Felix Made, Nohlanhla Tlotleng, Tahira Kootbodien, Vusi Ntlebi, Kerry Wilson, Nisha Naicker

**Affiliations:** 1 School of Health Systems and Public Health, University of Pretoria, Pretoria 0002, South Africa; 2 South African Field Epidemiology Training Program, National Institute for Communicable Diseases, National Health Laboratory Services, Johannesburg 2192, South Africa; 3 School of Public Health, University of Witwatersrand, Parktown 2000, South Africa; 4 Environmental Health Department, Faculty of Health Sciences, University of Johannesburg, Johannesburg 2028, South Africa; 5 Environment & Health Research Unit, South African Medical Research Council, Johannesburg 2012, South Africa; 6 National Institute of Occupational Health, National Health Laboratory Service, Braamfontein, Johannesburg 2001, South Africa

**Keywords:** common mental disorders, waste pickers, landfill sites

## Abstract

Waste-picking is an income-generating opportunity for individuals living in poverty. Waste picking is associated with a range of risk factors for common mental disorders (CMD). This study aimed to determine the prevalence and factors associated with CMD among waste pickers in Johannesburg. A cross-sectional study analyzed secondary data for 365 waste pickers. A validated Self-Reporting Questionnaire (SRQ-20) was used to assess CMD. Multivariable logistic regression was fitted to identify factors associated with CMD. The overall prevalence of CMD among waste pickers was 37.3%. The odds of having CMD were 2.5 and 3.2 higher in females and cigarette smokers, respectively (*p* = 0.019 and *p* = 0.003). Life enjoyment (Adjusted odds ratio [aOR] 0.54, *p* = 0.02) and a good quality of life (aOR 0.34, *p* ≤ 0.001) were associated with lower odds of CMD. The high prevalence of CMD among waste pickers was significantly associated with cigarette smoking, being female, not enjoying life, and a poor quality of life. Mental health awareness of CMD will assist with the prevention, early detection, and comprehensive management of CMD among waste pickers.

## 1. Introduction

Poverty and unemployment have driven many individuals to seek income opportunities in the informal economy [1,2]. Waste picking is a type of informal employment that occurs worldwide. Waste pickers reclaim and recycle waste from a variety of waste streams, primarily landfill sites, to sell or reuse [1]. In developing countries, there are approximately 15 million waste pickers. Regardless of their visibility and contribution to the environmental and economy sustainability in most cities, these waste pickers are overlooked [3]. Owing to the nature of their work, waste pickers may be exposed to a range of health hazards, including degraded food waste, sharp objects, and products containing toxic metals, such as lead and mercury [4,5]. In addition to physical hazards, waste pickers may experience psychological symptoms [1,2]. A study done in South Africa has highlighted the association between socioeconomic deprivation resulting from unemployment and various psychological disorders [5]. Informal waste pickers may also be subjected to social stigma and bullying or conflict while competing for waste resources [1,2,3,4]. Waste pickers often live in isolation as a result of stigma and may experience low self-esteem, which may negatively affect their psychological wellbeing [1].

### 1.1. Common Mental Disorders

Common mental disorders (CMD) are anxiety and depressive disorders. These disorders are categorized as neurotic, stress-related, mood, and somatoform disorders [6]. Depressive disorders can be recurrent or long-lasting and can impair one’s ability to function and cope with daily activities, such as work or school [6,7]. Depressive disorders are characterized by sadness, loss of interest in pleasurable activities, feelings of guilt, low self esteem, disturbed sleep or appetite, feelings of tiredness, and poor concentration. At its most severe, depression can lead to suicide [7]. Depressive disorders include two main sub-categories: major depressive disorder and dysthymia. Major depressive disorder is characterized by symptoms such as depressed mood, loss of interest and enjoyment, and decreased energy. Depending on the severity of the symptoms, a depressive episode can be categorized as mild, moderate, or severe. Dysthymia is a chronic form of mild depression. The symptoms of dysthymia are similar to a depressive episode but tend to be less extreme and last longer [7].

Anxiety disorders refer to a cluster of mental disorders characterized by feelings of anxiety and fear. These disorders includes generalised anxiety disorder (GAD), panic disorder, phobias, social anxiety disorder, obsessive-compulsive disorder (OCD), and post-traumatic stress disorder (PTSD). As with depression, symptoms can range from mild to severe. The duration of symptoms typically experienced by people with anxiety disorders makes it a more chronic than episodic disorder [7,8]. The prevalence of CMD is increasing globally, particularly in lower-income countries [7]. At a global level, over 300 million people are estimated to suffer from depression, which is equivalent to 4.4% of the world’s population [7]. In South Africa, it is estimated that about 30% of the population has life-long psychiatric disorders, and around 16% suffers from depression, anxiety, or substance use disorders [5,6,7,8]. Thus, one in three South Africans will be affected by mental illness in their lifetime [5].

### 1.2. The Well-Being of Waste Pickers

Informal employment is presumed to be a significant but rarely studied social determinant of health [9]. Several studies show compelling evidence that occupation may influence physical and mental wellbeing [10]. The workplace environment can have a significant impact, either positively or negatively, on an individual’s physical and mental wellbeing [10,11]. A study conducted in England on the contribution of work and non-work stress to CMD revealed that having a “poor quality”, or hated, job may have an impact on mental health [11]. Waste picking counts amongst those jobs that people engage in out of desperation and is usually of “poor-quality” [1,2]. A study conducted in South Africa has reported that unemployment and poverty, which are the main drivers of engagement in waste picking, have been linked to mental illness [12]. Shame, stigma, and humiliation are amongst the psychological impacts of living in poverty, which may negatively impact one’s psychological wellbeing [13]. A study on the psychological wellbeing of waste pickers in Mumbai reported that waste pickers had an unhealthy psychological state [1]. In addition, another study conducted in Brazil indicated that levels of CMD were higher in informal workers, relative to those who were formally employed [14]. The authors suggested that informal work may have adverse consequences on psychological health [1,4,10]. The lack of organization of the informal sector, as well as the vulnerability associated with having limited control over earnings, working conditions, and low earnings may have contributed to such adverse consequences, including an increase in the risk of anxiety and stress [15]. Information on the health issues and other challenges that waste pickers encounter is of high importance as the basis for interventions to address the determinants of CMD in this group.

### 1.3. The Present Study

There is limited research information on waste pickers’ mental health in South Africa and other African countries. This study aimed to assess the prevalence of CMD and factors associated with CMD in waste pickers at selected landfill sites in Johannesburg, South Africa.

## 2. Materials and Methods

### 2.1. Study Design and Setting

We conducted a cross-sectional analysis on secondary data from the 2017 National Institute for Occupational Health (NIOH) study. Briefly, the primary NIOH study surveyed waste pickers in two landfill sites in Johannesburg, South Africa to assess their health status and access to healthcare services. The study participants were informal waste pickers aged 18 years and older who were present on the day of, and volunteered to participate in, the survey. Trained field workers administered a structured questionnaire to each participant through face-to-face interviews using the language preferred by the participants. The questionnaire was made up of the following scales: socio-metric data: a series of open questions that collected information on personal variables of the participants, such as age, sex, education, area of residence, income, and the number of years working as a waste picker on any landfill site. Behavioral characteristics included current cigarette smoking and current alcohol use, as well as health outcomes, such as chronic ill health (hypertension, diabetes, tuberculosis (TB), and human immunodeficiency virus (HIV) status. In addition, a Self-Reporting Questionnaire (SRQ-20), a screening instrument for assessing mental disorders, was also administered to each participant.

### 2.2. Study Size and Sampling

Based on an estimated total population of 3600 waste recyclers at Johannesburg landfills, a survey sample size calculation indicated the need for a sample size of 365, with a confidence level of 95% and a significance level of 0.05. A total of 365 conveniently recruited participants completed the survey: 82% (*n* = 299) was from Site 1 and 17% (*n* = 62) from Site 2. However, 33 were excluded due to the provision of incomplete SRQ-20 information. Completion of all 20 questions in the SRQ-20 questionnaire is mandatory for the diagnosis of CMD. The response rate was 91%.

#### Self-Reporting Questionnaire (SRQ-20)

The SRQ-20 is a screening instrument used to assess mental disorders and was developed by the World Health Organization (WHO) in 1994 [16]. The SRQ-20 includes questions about feelings of sadness, physical symptoms of mental disorders, suicidal ideation during the four weeks prior to the interview, as well as effects on daily living activities (Table 1). This questionnaire consists of twenty yes/no questions. A score of >8 out of the twenty questions indicates that the participant has scored positive for CMD [16,17]. Since its inception, the SRQ-20 has been tested for its validity in developed and developing countries and has been previously used in South African studies [17]. The SRQ-20 was reported to have good internal consistency at (α = 0.84) and good sensitivity and specificity at 63% and 88%, respectively. The use of local languages and trained field workers ensured validity [16].

### 2.3. Operational Words

#### 2.3.1. CMD Positive

For each question that a participant answers “yes”, a mark is allocated. Participants who scored eight or more on the SRQ questions were classified as having CMD.

#### 2.3.2. Good Quality of Life

On a scale of one to five (1. very poor, 2. poor, 3. neither poor or good, 4. Good, and 5. very good), participants rated how they view their quality of life (QOL). All participants that gave a score between one and three were classified as having a poor QOL, while those that gave a score of four or five were classified as having good QOL.

#### 2.3.3. Enjoying Life

On a scale of one to five (1. not at all, 2. do not enjoy life, 3. neither enjoy life or not, 4. enjoy life, and 5. extremely enjoy life), participants rated how much they enjoy life. All participants that gave a score between one and three were classified as participants who do not enjoy life, while those that gave a score of four or five were classified as enjoying life.

### 2.4. Data Analysis

The data set from the 2018 National Institute for Occupational Health (NIOH) study was analyzed using Stata version 15 (StataCorp. 2017. Stata Statistical Software: Release 15. College Station, TX: StataCorp LP). Categorical variables were tabulated in absolute numbers and percentages. Continuous variables were summarized using medians. Differences in proportions between the groups were compared using the Pearson Chi-square test. Univariate and multivariable logistic regression analyses were used to determine associations between CMD and related factors. Variables with a *p*-value < 0.2 in the univariate analysis were included in the multivariable analysis. Variables with a *p*-value of less than 0.05 were retained in the final multivariable model. The Hosmer-Lemeshow test was used to assess the goodness of fit of the regression model. The odds ratios (ORs) and corresponding 95% confidence intervals (95% CIs) and *p*-values were tabulated.

### 2.5. Ethical Clearance

Ethical approval for the primary study was obtained from the Human Research Ethics Committee (Medical) of the University of Witwatersrand, South Africa (certificate number: M171120). The current study protocol was assessed and approved by the Faculty of Health Sciences Research Ethics Committee of the University of Pretoria (Ethic Number 41/2019).

## 3. Results

### 3.1. Demographics of Waste Pickers

Twenty-nine (29) study participants were excluded due to the provision of incomplete information for the SRQ-20 component of the survey questionnaire, which is mandatory for the diagnosis of CMD. With respect to the final sample (*n* = 332), the majority were male (241/72, 5%) and had secondary education (259/78.0%). The median age of participants was 31 (range 18–81). Approximately 155 (46.6%) participants were aged 18 to 30 years old. About 97 (29.5%) of the participants were residing adjacent to the landfill. Most (177/54.2%) of the participants had worked for less than five years. The majority of the participants reported that they consumed alcohol 258 (77.7%) and smoked cigarettes 222 (68.9%). The majority 270 (81.5%) were South African by birth and most 189 (57.6%) had an average monthly income of R3000 ($208.07) to R5000 ($346.78). With regard to chronic illness, HIV was the most prevalent at 38 (11.2%) (Supplementary: Table S1).

### 3.2. Prevalence of Common Mental Disorders among Waste Pickers

Table 2 lists the prevalence of CMD symptoms by factors that were significantly associated with CMD. The overall prevalence of CMD among informal waste pickers was 123 (37.5%). Site 1 had a higher 111 (40.8%) prevalence of CMD symptoms compared to site 12 (20.3%). The prevalence of CMD was significantly higher among participants who were residing adjacent to the landfill (*p* = 0.011), smoking cigarettes (*p* = 0.032), experiencing poor quality of life (*p* ≤ 0.001), or not enjoying life (*p* ≤ 0.001).

### 3.3. Factors Associated with Common Mental Disorders among Waste Pickers

Table 3 presents the results from the univariate and multivariable logistic regression analysis, giving the odds ratio adjusted for age and sex. The odds of having symptoms of CMD were twice as high in female waste pickers compared to their male counterparts (aOR 2.5, 95% CI: 1.16–5.54, *p* = 0.019) and three times higher among cigarette smokers compared to non-smokers (OR 3.2, 95% CI: 1.48–7.04, *p* = 0.003). The odds of having symptoms of CMD were lower among participants who reported life enjoyment (aOR 0.3, 95% CI: 0.12–0.23, *p* ≤ 0.001) and a good quality of life (aOR 0.5, 95% CI: 0.32–0.91, *p* = 0.02).

## 4. Discussion

The present study estimated the prevalence of CMD and associated risk factors by employing the SRQ-20 among informal waste pickers. The findings indicate that the overall prevalence of CMD among waste pickers (37.5%) was high compared to the prevalence of CMD in the general population of South Africa. A national survey done on the general population of South Africa in 2008 reported a CMD prevalence of 16.4% [5]. This could be attributed to the notion that waste pickers experience several important predictors of CMD in great magnitude [1]. Predictors, such as poverty and living and working in an unhealthy or dirty environment, have a negative impact on one’s psychological wellbeing [18]. Furthermore, psychological stressors, such as living in isolation and the stigma and bullying that occur amongst waste pickers as they compete for resources, psychologically affect waste pickers [1,2,11,18]. However, we cannot ignore the reverse causality that may occur between informal employment and a high prevalence of CMD. Waste pickers may have psychological ailments prior to taking up work in the waste picking sector [15].

### 4.1. CMD among Waste Pickers: Associated with Gender

This study indicates that females were more affected by symptoms of CMD than males. This is in agreement with several studies on CMD [1,2,3,4,5,6]. A study undertaken in India assessing the psychological wellbeing of waste pickers and a survey conducted in South Africa about the lifetime prevalence of psychiatric disorders both indicated that females were most affected by CMD relative to their male counterparts [1,2,5,12]. Similarly, a study undertaken in Brazil on informal work and common mental disorders indicated that females were more affected than males [15]. This could be attributed to the fact that female waste pickers possibly face more work stress than their male counterparts, in order to make an adequate income from waste picking. Women may also be at a physical disadvantage relative to men in the competition for waste resources at landfill sites [2,3]. Beyond the stressors of being a waste picker, being a female waste picker is associated with additional stress.

### 4.2. CMD among Waste Pickers: Associated with Cigarette Smoking

This study indicates that cigarette smoking was significantly associated with symptoms of CMD. Smoking is a well-documented predictor for the onset of CMD. Daily smoking has been linked to an increase in the first occurrence of panic disorders [19]. A study on the prevalence of smoking and mental illness reported that persons with mental illness were twice as likely to smoke [20]. As a waste picker, negative emotions resulting from bullying and stigma are encountered on a daily basis. In response, smoking may be taken up as a coping mechanism to help alleviate negative emotions [19,20].

### 4.3. CMD among Waste Pickers: Associated Good Quality of Life and Enjoying Life

In this study, enjoying life and having a good quality of life had a protective effect against having any CMD. A study undertaken in New York on the health-related quality of life in primary care patients with mental disorders indicated that mental disorders commonly seen in primary care are not only associated with medical disorders but more so with impairment of quality of life [21]. Waguih et al. [22] reported that patients with major depressive disorders had a significantly low quality of life [22]. Another study on quality of life and mental disorders conducted in Europe showed that patients with severe Generalized Anxiety Disorder (GAD) had an impaired quality of life and, consequently, those with less severe GAD had a better quality of life [23]. Waste pickers generally perceive themselves as having a poor quality of life due to the type of environment they live in and the nature of their job. This perception has a negative impact on their emotions. However, participants who, despite living and working in the same conditions, regarded their quality of life as being good had better mental health outcomes.

### 4.4. Limitations

Amongst the limitations of this study are that the results may not be generalizable to the South African population as a whole, as the parent study was only undertaken in two selected landfills sites. Participants in the parent survey were selected though convenient sampling methods, and. Therefore, may not truly represent the entire community of waste pickers. The use of self-reported questionnaires on aspects related to mental health could be affected by a positivist bias or a tendency to overestimate the real values of mental health, as well as recall bias. Due to the cross-sectional nature of the study’s design, it is difficult to draw a conclusion about the causality between CMD and its associated risk factors.

## 5. Conclusions

The findings of the study indicate that the prevalence of CMD among waste pickers in the study settings in Johannesburg is high and associated particularly with being female, a smoker, and having a poor quality of life. People with CMD experience significant limitation in functioning at the personal and societal levels, a consequence of which may be an escalation in the mental health treatment burden for the public health sector in South Africa. Formalizing the waste picking industry may assist in bringing structure and security to this group—increasing safety, especially for female waste pickers. Health education on the effects of smoking on mental health and mental health awareness programs will allow waste pickers to identify key symptoms of CMD at an early stage. This may also assist with the prevention, early detection, and comprehensive management of CMD among waste pickers.

## Figures and Tables

**Table 1:** Self-Reporting Questionnaire (SRQ-20) 20 items. CMD, common mental disorders.

Number	Questions to Assess CMD	Yes	No
1	Do you often have headaches?	Yes	No
2	Is your appetite poor?	Yes	No
3	Do you sleep badly?	Yes	No
4	Are you easily frightened?	Yes	No
5	Do your hands shake?	Yes	No
6	Do you feel nervous, tense or worried?	Yes	No
7	Is your digestion poor?	Yes	No
8	Do you have trouble thinking clearly?	Yes	No
9	Do you feel unhappy?	Yes	No
10	Do you cry more than usual?	Yes	No
11	Do you find it difficult to enjoy your daily activities?	Yes	No
12	Do you find it difficult to make decisions?	Yes	No
13	Is your daily work suffering?	Yes	No
14	Are you unable to play a useful part in life?	Yes	No
15	Have you lost interest in things?	Yes	No
16	Do you feel that you are a worthless person?	Yes	No
17	Has the thought of ending your life been on your mind?	Yes	No
18	Do you feel tired all the time?	Yes	No
19	Do you have uncomfortable feelings in your stomach?	Yes	No
20	Are you tired easily?	Yes	No
	Total number of yes answers		

**Table 2:** Prevalence of CMD by socio-demographic factors among waste pickers.

Socio-Demographic Factors	*N* = 332, Missing (*n*)	No CMD (*N*, %)	CMD (*N*, %)	*p*-Value
**Sex**				
Male	241	152 (73.08)	89 (71.77)	
Female	91	56 (26.92)	35 (28.23)	0.797
**Ages**				
18–30	155	92 (44.23)	63 (50.81)	
31–40	108	70 (33.65)	38 (30.65)	
41–50	40	29 (13.94)	11 (8.87)	
51+	29	17 (8.17)	12 (9.68)	0.424
**Education**				
None	13	11 (5.29)	2 (1.61)	
Primary	55	37 (17.79)	18 (14.52)	
Secondary	289	157 (75.08)	102 (82.26)	
Tertiary	5	3 (1.49)	2 (1.61)	0.301
**Living adjacent to the landfill**				
Yes	231	50 (52.08)	46 (47.92)	
No	96	155 (67.10)	76 (32.90)	0.011
	(5)			
**Smoking history**				
Yes	222	129 (58.11)	93 (41.89)	
No	99	70 (70.71	29 (29.29)	0.032
	(11)			
**Alcohol cosumption**				
Yes	258	163 (70.37)	29 (23.39)	
No	75	45 (21.68)	95 (76.61)	0.711
**Quality of life (QOL)**				
Good QOL	172	124 (72.09)	481(27.91)	
Poor QOL	155	82 (52.90)	73 (47.10)	<0.001
	(5)			
**Life enjoyment**				
Enjoy life	182	135 (74.18)	47 (25.82)	
Do not enjoy life	147	72 (48.98)	75 (51.02)	<0.001
	(3)			

**Table 3:** Univariate and Multivariable analysis of the predictors of common mental disorders among informal waste Pickers.

Characteristics	Univariate Analysis			Multivariable Analysis		
	Odd Ratio	95% CI	*p*-Value	Odds Ratio	95% CI	*p*-Value
**Sex**						
Males						
Females	1.067416	0.64–1.75	0.79			
**Age**						
18–30						
31–40	0.7927438	0.47–1.31	0.371			
41–50	0.5539135	0.25–1.18	1.130			
51+	1.030812	0.46–2.30	0.941			
**Alcohol use**						
No						
Yes	0.904379	0.53–1.53	0.711			
**Living adjacent to landfill**						
No						
Yes	1.917105	1.81–3.10	0.008 *	1.64	0.95–2.82	0.071
**Current smoker**						
No						
Yes	1.682171	1.01–2.78	0.04 *	3.233811	1.48–7.04	0.003 *
**Quality of life**						
Poor QOL						
Good QOL	0.3793488	0.23–0.60	<0.001 *	0.5461238	0.32–0.91	0.02 *
**Enjoying life**						
No						
Yes	0.3298246	0.20–0.52	<0.001 *	0.3490361	0.12–0.23	<0.001 *

Multivariable analysis; Adjusted for age and gender. * *p* < 0.05.

## Supplementary Materials

The following are available online at https://www.mdpi.com/1660-4601/16/14/2618/s1, Table S1: Distribution of socio-demographic features of the waste pickers in Johannesburg; File S1: Primary study questionnaire: Occupational hazards and health access of waste pickers in Johannesburg, South Africa.
