# Supplementary material for: Common Mental Health Disorders among Informal Waste Pickers in Johannesburg, South Africa 2018—A Cross-Sectional Study
Source: Int J Environ Res Public Health. 2019 Jul 23;16(14):2618. doi: 10.3390/ijerph16142618 (PMC6678252; doi:10.3390/ijerph16142618)
Supplement: Supplementary file 1 [file ijerph-16-02618-s001.zip › ijerph-541486-supp. Proofed/ijerph-541486- File S1.docx]

**Supplementary material:**

File S1: Primary study questionnaire: Occupational hazards and health access of waste pickers in Johannesburg, South Africa

Health and health care access of landfill waste pickers

Date of Interview: 2018 / mm / dd

Interviewer: ………………………………………………………………………………………….

Name of land fill site: ………………………………………………………………………………

1. **Demographic and household Information**
2. Age
3. Gender: M / F
4. Main language spoken (choose one)

| English |  |
| --- | --- |
| Afrikaans |  |
| Zulu |  |
| Xhosa |  |
| Ndebele |  |
| Sepedi |  |
| Sesotho |  |
| Setswana |  |
| Tsonga |  |
| Swati |  |
| Venda |  |
| Other. Please specify…………………………. |  |

1. What is the last grade that you completed?

Indicate if:

4.1 No schooling? Y / N

4.2 Tertiary level completed? Y / N

1. Country of birth

5.1 If not South African, how many years have you been living in SA?

1. Province of birth

6.1 How long have you lived in Johannesburg?

6.2 Where did you live before you moved to Johannesburg?

6.3 Why did you move to Johannesburg?

1. Do you live on or adjacent to a landfill site? Y / N
2. Do you live in a: (choose one)?

| Formal house |  |
| --- | --- |
| Informal dwelling |  |
| Back yard dwelling / room - formal |  |
| Back yard dwelling / room - informal |  |
| Other (e.g. veld, bushes, street) |  |

1. What do you mainly use for cooking at home? (choose one)

| Electricity |  |
| --- | --- |
| Paraffin |  |
| Gas |  |
| Wood |  |
| Coal |  |
| Other |  |

1. What do you mainly use for heating at home? (choose one)

| Electricity |  |
| --- | --- |
| Paraffin |  |
| Gas |  |
| Wood |  |
| Coal |  |
| Other |  |

1. Where do you mainly get your water from at home? (choose one)

| River / stream |  |
| --- | --- |
| Household tap |  |
| Communal tap |  |
| Borehole |  |
| Other |  |

1. Do you have access to toilet facilities where you live? Y / N

12.1 If yes, what type of toilet?

| Private flush toilet |  |
| --- | --- |
| Communal flush toilet |  |
| Pit latrine |  |
| Chemical toilet |  |
| Other |  |

1. Do you have access to a place to wash yourself/ hands at home? Y / N
2. How many people are there in your household?

14.1 Adults (15 years and older)

14.2 Children (14 years and younger)

1. Are you the sole bread winner for your household? Y / N
2. How many people do you support financially?
3. What is your average monthly income?
4. Do you receive any grants? Y / N

| pension | disability | child support |
| --- | --- | --- |

18.1 If yes, please indicate

1. How much money do you spend on the following items every month?

| 1. Food | R |
| --- | --- |
| 2. Transport (include taxi fare, petrol, car instalment) | R |
| 3. Transport of waste to where you sell your waste e.g. recycling plant | R |
| 4. Housing- rent, bond etc | R |
| 5. Water | R |
| 6. Electricity | R |
| 7. Paraffin | R |
| 8. Coal | R |
| 9. Wood | R |
| 10. Gas | R |
| 11. Other type of fuel: Specify ………………………………… | R |
| 12. Alcohol | R |
| 13. Cigarettes | R |
| 14. Telephones & Cellular Telephones | R |
| 15. Medical expenses (include transport to clinics, medication etc) | R |
| 16. Schooling or university (including uniforms, books, fees etc) | R |
| 17. Debt repayments | R |
| 18. Entertainment e.g. movies etc. Please specify………………………………………. | R |

1. Where do you get your food from? (choose all that apply)

| Grow your own food |  |
| --- | --- |
| Buy food |  |
| From charities |  |
| From waste pickers |  |
| From landfill site |  |
| Other …………………………………………. |  |

1. Food security (Community Childhood Hunger Identification Project Index – CCHIP)

| **Household level food insecurity** |  |
| --- | --- |
| 21.1 Does your household ever run out of money to buy food?  a. in the past 30 days?  b. 5 or more days in the past 30 days? | Y / N  Y / N |
| 21.2 Do you ever rely on a limited number of foods to feed your children because you are running out of money to buy food for a meal?  a. in the past 30 days?  b. 5 or more days in the past 30 days? | Y / N  Y / N |
| 21.3 Do you ever cut the size of meals or skip meals because there is not enough money for food?  a. in the past 30 days?  b. 5 or more days in the past 30 days? | Y / N  Y / N |
| **Individual-level food insecurity** |  |
| 21.4 Do you ever eat less than you should because there is not enough money for food?  a. in the past 30 days?  b. 5 or more days in the past 30 days? | Y / N  Y / N |
| **Child hunger** |  |
| 21.5 Do your children ever eat less than you feel they should because there is not enough money?  a. in the past 30 days?  b. 5 or more days in the past 30 days? | Y / N  Y / N |
| 21.6 Do your children ever say they are hungry because there is not enough food in the house?  a. in the past 30 days?  b. 5 or more days in the past 30 days? | Y / N  Y / N |
| 21.7 Do you ever cut the size of your children’s meals or do they ever skip meals because there is not enough money to buy food?  a. in the past 30 days?  b. 5 or more days in the past 30 days? | Y / N  Y / N |
| 21.8 Do any of your children ever go to bed because there is not enough money to buy food?  a. in the past 30 days?  b. 5 or more days in the past 30 days? | Y / N  Y / N |

1. **Occupational history**
2. Apart from collecting recyclable material and getting grants, do you get money from any other sources? Y/N

22.1 If yes, what are these other sources of income and how much do you get from them per month?

| **Source of income** | **Amount (Rands) [monthly]** |
| --- | --- |
| a. |  |
| b. |  |
| c. |  |

1. Before working as a waste picker, what was your previous type of employment/s? ..…………………………………………………………………………………………………..
2. What was the reason for leaving your last job? ………………………………………….
3. For how many years have you worked as a waste picker? ………………………………..
4. How many days per week do you work on the landfill? ………………………….
5. How many hours per day are you on the landfill? ……………………………………………
6. Please describe the days of the week and the times that you work.

| **Days of the week** | **Start time** | **End time** |
| --- | --- | --- |
| Monday |  |  |
| Tuesday |  |  |
| Wednesday |  |  |
| Thursday |  |  |
| Friday |  |  |
| Saturday |  |  |
| Sunday |  |  |

1. **Waste Picking**
2. Why did you choose waste picking?

………………………………………………………………………………………………………………………………………………………………………………………………………………

1. What do you like about waste picking?

………………………………………………………………………………………………………………………………………………………………………………………………………………

1. What don’t you like about waste picking?

………………………………………………………………………………………………………………………………………………………………………………………………………………………………………………………………………………………………………………………………………

1. Do you collect any of the following waste? (choose all that apply)

| Paper, cardboard | Plastic | Metals, cans | Electronic waste | glass | cloth | Other  ………………………. |
| --- | --- | --- | --- | --- | --- | --- |

1. If you collect more than one type of material, which material do you collect the most of? (choose only one)

| Paper, cardboard | Plastic | Metals, cans | Electronic waste | glass | cloth | Other  ………………………. |
| --- | --- | --- | --- | --- | --- | --- |

1. Do you only collect one type of waste per day or mixed? E.g. paper on Monday and metal on Tuesday or mixed every day. Y / N
2. How much money do you earn from waste picking on average?

35.1 on a good day?

35.2 on a bad day?

1. What amount of waste do you remove on average per day, using a refuse black bag as a reference? e.g. 2 full black bags, half a black bag etc.

..……………………………………………………………………………………………………

1. How much does it weigh on average per day? …………………………………………...kg
2. How do you transport your materials to the next location? ………………………………….
3. How often do you take the materials to sell? (choose one)

| Daily | 2 x weekly | Weekly | 2 weeks | Month | Other (please specify)  ………………………. |
| --- | --- | --- | --- | --- | --- |

1. Where do you take your waste to sell?

| Directly to the recycling centre | To buyers or dealers who will sell your waste to the recycling centre | Other (please specify)  ………………………. |
| --- | --- | --- |

1. If you don’t sell your waste every day, where do you store the waste you have collected?

| At home | On this landfill | Another landfill | Other (please specify)  ………………………. |
| --- | --- | --- | --- |

1. **PPE**
2. Do you have access to toilet facilities at the landfill? Y / N

42.1 If no, what do you do? …………………………………………………………………..

1. Do you think washing hands is important? Y / N
2. Do you have access to water on the landfill? Y / N
3. Do you eat on the landfill while working on the landfill? Y / N
4. Do you wash your hands?

46.1 Before eating while you are working at the landfill site? Y / N

46.2 At the end of the day, after working? Y / N

1. What do you use to wash your hands? (choose all that apply)

| Water |  |
| --- | --- |
| Water and Soap |  |
| Other…………………………… |  |

1. Do you have or own:

| Masks | Y / N |
| --- | --- |
| Gloves | Y / N |
| Boots or closed shoes | Y / N |
| Other…………………………… |  |

48.1 If yes, where did you get it from?.................................................................................

48.2 Do you wear it while working? (choose one)

| Always |  |
| --- | --- |
| Sometimes |  |
| Never |  |

48.3 If no, why not………………………………………………………………………………

1. **Occupational exposures**
2. Do you lift heavy objects while working on the landfill? Y / N
3. Have you ever been cut by the materials you handle on the landfill site? Y / N
4. Have you ever handled needles on the landfill site? Y / N
5. Have you ever been injured by a needle on the landfill site? Y / N
6. Have you ever handled blood on the landfill site? Y / N
7. Are there dogs on the landfill site? Y / N
8. Have you ever been attacked by a dog on the landfill site? Y / N
9. Have you seen rats/mice on the landfill site? Y / N
10. Have you ever been bitten by rodents on the landfill site? Y / N
11. How problematic are mosquitoes on the landfill site?

| No problem |  |
| --- | --- |
| Moderate problem |  |
| Major problem |  |

1. How problematic is airborne dust on the landfill site?

| No problem |  |
| --- | --- |
| Moderate problem |  |
| Major problem |  |

1. How problematic are strong gas smells on the landfill site?

| No problem |  |
| --- | --- |
| Moderate problem |  |
| Major problem |  |

1. Are there pools of dirty water on the surface of the landfill site? Y / N
2. Have you ever had waste fall on top of you? Y / N
3. Have you ever been hurt by waste falling on you? Y / N
4. Have you ever hurt yourself by falling on the landfill site? Y / N
5. Have you ever see a fire on the landfill site? Y / N
6. Have you ever seen an explosion on the landfill site? Y / N
7. Have you ever been injured by any type of vehicle on the landfill site? Y / N
8. Have you ever handled paint on the landfill site? Y / N
9. Have you ever handled chemicals, such as detergents or anything that smells very strong? Y / N
10. Have you ever been injured during violence involving another waste picker? Y / N
11. Have you ever been injured during violence involving security guards? Y / N
12. What 3 or more things worry you the most about working on the landfill?

……………………………………………………………………………………………………………………………………………………………………………………………………………………………………………………………………………………………………………………………………………………………………………………………………………………………………………………

1. Do you think that the landfill site is a safe place to work? Y / N / Not sure

Please explain

……………………………………………………………………………………………………………………………………………………………………………………………………………………………………………………………………………………………………………………………………………………………………………………………………………………………………………………

1. Do you experience any problems accessing the landfill site? Y / N

74.1 If yes, please explain…………………………………………………………………………….

…………………………………………………………………………………………………………………………………………………………………………………………………………………………

1. How do security guards at the landfill treat you?

…………………………………………………………………………………………………………………………………………………………………………………………………………………………

1. Do you belong to any waste picker forum? Y / N

76.1 If yes, in what ways does belonging to the forum help you?

…………………………………………………………………………………………………………………………………………………………………………………………………………………………

1. **Health**
2. Do you smoke currently? Y/ N

77.1 If no, have you ever smoked? Y / N

77.2 If yes, what do you smoke?

| Cigarettes | Snuff | Chewing tobacco | Dagga/  weed | Hookah | Other |
| --- | --- | --- | --- | --- | --- |

77.3 How much do you smoke/chew or snuff per day? ………………

77.4 For how many years have you smoked? ……………………………….

1. Alcohol Use Disorder Identification Test (AUDIT)

| ***Instructions for interviewer: W****e would like to ask you questions about your use of alcohol like beer, wine, brandy, whiskey or vodka during this past year.* | |
| --- | --- |
| **78.1. How often do you have a drink**  **containing alcohol?**  (0) Never (Skip to Qs 9 -10)  (1) Monthly or less  (2) 2 to 4 times a month  (3) 2 to 3 times a week  (4) 4 or more times a week | **78.6. How often during the last year have you**  **needed a first drink in the morning to get**  **yourself going after a heavy drinking**  **session?**  (0) Never  (1) Less than monthly  (2) Monthly  (3) Weekly  (4) Daily or almost daily |
| **78.2. How many drinks containing alcohol do**  **you have on a typical day when you are**  **drinking?**  (0) 1 or 2  (1) 3 or 4  (2) 5 or 6  (3) 7, 8 or 9  (4) 10 or more | **78.7. How often during the last year have you**  **had a feeling of guilt or remorse after**  **drinking?**  (0) Never  (1) Less than monthly  (2) Monthly  (3) Weekly  (4) Daily or almost daily |
| **78.3 How often do you have six or more drinks**  **on one occasion?**  (0) Never  (1) Less than monthly  (2) Monthly  (3) Weekly  (4) Daily or almost daily | **78.8. How often during the last year have you**  **been unable to remember what happened**  **the night before because you had been**  **drinking?**  (0) Never  (1) Less than monthly  (2) Monthly  (3) Weekly  (4) Daily or almost daily |
| **78.4. How often during the last year have you**  **found that you were not able to stop**  **drinking once you had started?**  (0) Never  (1) Less than monthly  (2) Monthly  (3) Weekly  (4) Daily or almost daily | **78.9. Have you or someone else been injured**  **as a result of your drinking?**  (0) No  (2) Yes, but not in the last year  (4) Yes, during the last year |
| **78.5. How often during the last year have you**  **failed to do what was normally expected**  **from you because of drinking ?**  (0) Never  (1) Less than monthly  (2) Monthly  (3) Weekly  (4) Daily or almost daily | **78.10. Has a relative or friend or a doctor or**  **another health worker been concerned**  **about your drinking?**  (0) No  (2) Yes, but not in the last year  (4) Yes, during the last year |
| Record total of specific items here ……………... | |

1. In general, would you say that your health is

| excellent | Very good | Good | fair | poor |
| --- | --- | --- | --- | --- |

79.1 If fair or poor, please explain…………………………………………………………..

1. **Acute symptoms:** In the past 2 weeks, have you experienced any of the following symptoms (choose any that apply)

| **Acute symptom** | | If yes, did you seek medical treatment? | Who provided the treatment? |
| --- | --- | --- | --- |
| 80.1 Cough | Y / N | Y / N | 1. Clinic  2. Doctor  3. Traditional healer  4. Self-medicated  5. Other ………………….. |
| 80.2 Itchy rash | Y / N | Y / N | 1. Clinic  2. Doctor  3. Traditional healer  4. Self-medicated  5. Other ………………….. |
| 80.3 Runny/blocked nose | Y / N | Y / N | 1. Clinic  2. Doctor  3. Traditional healer  4. Self-medicated  5. Other ………………….. |
| 80.4Teary/watery eyes | Y / N | Y / N | 1. Clinic  2. Doctor  3. Traditional healer  4. Self-medicated  5. Other ………………….. |
| 80.5 Sneezing | Y / N | Y / N | 1. Clinic  2. Doctor  3. Traditional healer  4. Self-medicated  5. Other ………………….. |
| 80.6 Breathlessness | Y / N | Y / N | 1. Clinic  2. Doctor  3. Traditional healer  4. Self-medicated  5. Other ………………….. |
| 80.7 Rapid breathing | Y / N | Y / N | 1. Clinic  2. Doctor  3. Traditional healer  4. Self-medicated  5. Other ………………….. |
| 80.8 Rapid heart rate | Y / N | Y / N | 1. Clinic  2. Doctor  3. Traditional healer  4. Self-medicated  5. Other ………………….. |
| 80.9 Nausea/ vomiting | Y / N | Y / N | 1. Clinic  2. Doctor  3. Traditional healer  4. Self-medicated  5. Other ………………….. |
| 80.10 Diarrhoea | Y / N | Y / N | 1. Clinic  2. Doctor  3. Traditional healer  4. Self-medicated  5. Other ………………….. |
| 80.11 Headache | Y / N | Y / N | 1. Clinic  2. Doctor  3. Traditional healer  4. Self-medicated  5. Other ………………….. |
| 80.12 Loss of coordination | Y / N | Y / N | 1. Clinic  2. Doctor  3. Traditional healer  4. Self-medicated  5. Other ………………….. |
| 80.13 Fever | Y / N | Y / N | 1. Clinic  2. Doctor  3. Traditional healer  4. Self-medicated  5. Other ………………….. |
| 80.14 Muscle aches | Y / N | Y / N | 1. Clinic  2. Doctor  3. Traditional healer  4. Self-medicated  5. Other ………………….. |
| 80.15 Dizziness | Y / N | Y / N | 1. Clinic  2. Doctor  3. Traditional healer  4. Self-medicated  5. Other ………………….. |
| 80.16 Sunburn | Y / N | Y / N | 1. Clinic  2. Doctor  3. Traditional healer  4. Self-medicated  5. Other ………………….. |

1. **Chronic illness:** Do you have now, or have you ever been diagnosed with any of the following?

| Chronic illness | | If yes, did you seek medical treatment? | Who provided the treatment? |
| --- | --- | --- | --- |
| 81.1 Diabetes | Y / N | Y / N | 1. Clinic  2. Doctor  3. Traditional healer  4. Self-medicated  5. Other ………………….. |
| 81.2 Hypertension | Y / N | Y / N | 1. Clinic  2. Doctor  3. Traditional healer  4. Self-medicated  5. Other ………………….. |
| 81.3 Stroke | Y / N | Y / N | 1. Clinic  2. Doctor  3. Traditional healer  4. Self-medicated  5. Other ………………….. |
| 81.4 Asthma | Y / N | Y / N | 1. Clinic  2. Doctor  3. Traditional healer  4. Self-medicated  5. Other ………………….. |
| 81.5 Problems with vision | Y / N | Y / N | 1. Clinic  2. Doctor  3. Traditional healer  4. Self-medicated  5. Other ………………….. |
| 81.6 Problems with hearing | Y / N | Y / N | 1. Clinic  2. Doctor  3. Traditional healer  4. Self-medicated  5. Other ………………….. |
| 81.7 HIV | Y / N | Y / N | 1. Clinic  2. Doctor  3. Traditional healer  4. Self-medicated  5. Other ………………….. |
| 81.8 TB | Y / N | Y / N | 1. Clinic  2. Doctor  3. Traditional healer  4. Self-medicated  5. Other ………………….. |
| 81.9 Mental illness  Please specify  …………………………… | Y / N | Y / N | 1. Clinic  2. Doctor  3. Traditional healer  4. Self-medicated  5. Other ………………….. |
| 81.10 Cancer  Please specify  …………………………… | Y / N | Y / N | 1. Clinic  2. Doctor  3. Traditional healer  4. Self-medicated  5. Other ………………….. |
| 81.11 Disability type  Please specify  …………………………… | Y / N | Y / N | 1. Clinic  2. Doctor  3. Traditional healer  4. Self-medicated  5. Other ………………….. |

**Respiratory Symptoms (in past 12 months)**

1. Have you had a persistent cough for more than three weeks? Y/ N
2. How long has your cough lasted? Months ______ years_______
3. Have you ever had wheezing or whistling in the chest in the last 12 months? Y / N
4. How many attacks did you have in the last 12 months?..................................................
5. Did you seek medical treatment? Y / N

60.1 If yes, where did you go?

| Clinic | Hospital | Doctor | Traditional healer | Self-medicated | Other  …………… |
| --- | --- | --- | --- | --- | --- |

1. What was the diagnosis? ………………………………………………………………………..
2. Are there times when something at the landfill in the environment affects your chest? Y / N

88.1 If yes, how often have you experienced this? (Choose one)

| Every day |  |
| --- | --- |
| Several times a week |  |
| About once a week |  |
| Once or twice a month |  |
| A few times in the past 6 months |  |

88.2 Do you know what it is? ……………………………………………………………………

**Skin rashes**

1. Have you had an itchy rash that was coming and going for at least 6 months? Y /N

89.1 Have you had this rash any time in the last 6 months?

89.2 Has this rash affected

- 1. Folds of elbow Y / N
- 2. Behind knees Y / N
- 3. In front of ankles Y / N
- 4. Under buttocks Y / N
- 5. Around neck, ears or eyes Y / N

89.3 Did you seek medical treatment for your skin rash? Y / N

89.4 Where did you go?

| Clinic | Hospital | Doctor | Traditional healer | Self-medicated | Other  …………… |
| --- | --- | --- | --- | --- | --- |

89.5 What was the diagnosis?.............................................................................................

89.6 Have you ever been diagnosed with eczema by a doctor or nurse? Y / N

**Diarrhoea (in the last 6 months)**

1. Have you experienced diarrhoea, nausea or vomiting? Y / N

90.1 If yes, how often in the past 6 months have you experienced this? (Choose one)

| Every day |  |
| --- | --- |
| Several times a week |  |
| About once a week |  |
| Once or twice a month |  |
| A few times in the past 6 months |  |

90.2 Did you seek medical treatment? Y / N

90.3 Who provided the medical treatment?

| Clinic | Doctor | Traditional healer | Self-medicated | Other  …………………………… |
| --- | --- | --- | --- | --- |

**Injuries (in the last 6 months)**

1. Have you ever been injured at work (e.g. cuts, fractures, sprains and/or burns) Y / N

If yes, please specify the type of injury.

| **Type of injury** | | **If yes, how often did it occur?** (choose one) | **Did you receive first aid?** | **Who provided treatment?** |
| --- | --- | --- | --- | --- |
| 91.1 Fractures | Y / N | 1. Everyday  2. Several times a week  3. About once a month  4. Once or twice a month  5. A few times in the past 6 months | Y / N | 1. Pikitup staff  2. Clinic  3. Doctor  4.Tradtional healer  5. Self-medicated  6. Other……… |
| 91.2 Sprains and muscle strains | Y / N | 1. Everyday  2. Several times a week  3. About once a month  4. Once or twice a month  5. A few times in the past 6 months | Y / N | 1. Pikitup staff  2. Clinic  3. Doctor  4.Tradtional healer  5. Self-medicated  6. Other……… |
| 91.3 Injured by the landfill trucks | Y / N | 1. Everyday  2. Several times a week  3. About once a month  4. Once or twice a month  5. A few times in the past 6 months | Y / N | 1. Pikitup staff  2. Clinic  3. Doctor  4.Tradtional healer  5. Self-medicated  6. Other……… |
| 91.4 Burns | Y / N | 1. Everyday  2. Several times a week  3. About once a month  4. Once or twice a month  5. A few times in the past 6 months | Y / N | 1. Pikitup staff  2. Clinic  3. Doctor  4.Tradtional healer  5. Self-medicated  6. Other……… |
| 91.5 Being hit by falling objects | Y / N | 1. Everyday  2. Several times a week  3. About once a month  4. Once or twice a month  5. A few times in the past 6 months | Y / N | 1. Pikitup staff  2. Clinic  3. Doctor  4.Tradtional healer  5. Self-medicated  6. Other……… |
| 91.6 Slips, trips and falls | Y / N | 1. Everyday  2. Several times a week  3. About once a month  4. Once or twice a month  5. A few times in the past 6 months | Y / N | 1. Pikitup staff  2. Clinic  3. Doctor  4.Tradtional healer  5. Self-medicated  6. Other……… |
| 91.7 Cuts and lacerations | Y / N | 1. Everyday  2. Several times a week  3. About once a month  4. Once or twice a month  5. A few times in the past 6 months | Y / N | 1. Pikitup staff  2. Clinic  3. Doctor  4.Tradtional healer  5. Self-medicated  6. Other……… |
| 91.8 Inhaling toxic fumes | Y / N | 1. Everyday  2. Several times a week  3. About once a month  4. Once or twice a month  5. A few times in the past 6 months | Y / N | 1. Pikitup staff  2. Clinic  3. Doctor  4.Tradtional healer  5. Self-medicated  6. Other……… |
| 91.9 Exposure to **sudden** loud noise | Y / N | 1. Everyday  2. Several times a week  3. About once a month  4. Once or twice a month  5. A few times in the past 6 months | Y / N | 1. Pikitup staff  2. Clinic  3. Doctor  4.Tradtional healer  5. Self-medicated  6. Other……… |
| 91.10 Other  ……………………….. | Y / N | 1. Everyday  2. Several times a week  3. About once a month  4. Once or twice a month  5. A few times in the past 6 months | Y / N | 1. Pikitup staff  2. Clinic  3. Doctor  4.Tradtional healer  5. Self-medicated  6. Other……… |

1. If yes to 91.1 (fractures), 91.3 (injury from landfill trucks) or 91.4 (burns), please describe how you sustained your injury or injuries?

……………………………………………………………………………………………………….

……………………………………………………………………………………………………….

**Musculoskeletal Disorders (in the past 12 months)**

1. Have you suffered from any of the following? (choose any that apply)

| Back pain | Joint pain | Stiffness | Other  …………………………… |
| --- | --- | --- | --- |

93.1 If yes, did you seek medical treatment? Y / N

93.2 Who provided the medical treatment?

| Clinic | Doctor | Traditional healer | Self-medicated | Other  …………………………… |
| --- | --- | --- | --- | --- |

**Hearing assessment**

1. Do you have difficulty hearing or understanding people around you? Y / N

94.1 If yes, did you have a hearing problem before working on the landfill? Y / N

1. Is there a history of hearing problems or deafness in your family? Y / N
2. Have you ever been diagnosed with:

| Ear infection | Y / N |
| --- | --- |
| Meningitis | Y / N |
| TB | Y / N |
| Mumps / measles | Y / N |

**Serious Illness and injury (in past 12 months)**

1. Have you missed work due to any illness or injury? Y / N

97.1 If yes, please specify ….………………………………………………............................

97.2 Do you think your illness or injury was related to your work? Y / N

1. If yes, please give details……………………………………………………………………

**Reproductive health (Women only)**

1. How many times have you been pregnant? ........……………………………………………
2. How many children do you have? ……………………………………….……………….
3. Have you ever had a miscarriage? Y / N

101.1 If yes, when did it happen?

- Before working as a waste picker? Y/ N
- While working as a waste picker? Y / N

101.2 If yes, how many miscarriages did you have? …………………………………………..

1. Have you ever had a still birth? Y / N

102.1 If yes, when did it happen?

- Before working as a waste picker? Y/ N
- While working as a waste picker? Y / N

102.2 If yes, how many? ……………………………………………………………..

1. In your *last / most recent* pregnancy

103.1 Was this pregnancy expected or not a surprise? Y / N

103.2 How long did it take you to fall pregnant? (Years /months) ________ don’t know

103.3 Were you using contraceptives? Y / N

103.4 If yes, when did you stop your contraceptives?

(Years /months) ________ don’t know

103.5 Why did you stop your contraceptives?

| Fell pregnant | Side effects | cost | religion | N/A | Other  ………………… |
| --- | --- | --- | --- | --- | --- |

103.6 What was the outcome of this pregnancy?

| miscarriage | still birth | live birth |
| --- | --- | --- |

103.7 How many weeks were you when you delivered your last baby? ___________

103.8 What was the birth weight of your most recent child? ___________kg

1. **Mental health screening:**

*Interviewer: The following questions are related to certain pains and problems that may have bothered you in the last 30 days. If you think the question applies to you and you had the listed problem in the last 30 days, answer* ***YES****. On the other hand, if the question does not apply to you and you did not have the problem in the last 30 days, answer* ***NO.***

*Please do not discuss the following questions with anyone while answering. If you are unsure about how to answer a question, please give the best answer you can.*

*We would like to reassure you that the answers you are going to provide here are confidential.*

| **1** | Do you often have headaches? | Yes | No |
| --- | --- | --- | --- |
| **2** | Is your appetite poor? | Yes | No |
| **3** | Do you sleep badly? | Yes | No |
| **4** | Are you easily frightened? | Yes | No |
| **5** | Do your hands shake? | Yes | No |
| **6** | Do you feel nervous, tense or worried? | Yes | No |
| **7** | Is your digestion poor? | Yes | No |
| **8** | Do you have trouble thinking clearly? | Yes | No |
| **9** | Do you feel unhappy? | Yes | No |
| **10** | Do you cry more than usual? | Yes | No |
| **11** | Do you find it difficult to enjoy your daily activities? | Yes | No |
| **12** | Do you find it difficult to make decisions? | Yes | No |
| **13** | Is your daily work suffering? | Yes | No |
| **14** | Are you unable to play a useful part in life? | Yes | No |
| **15** | Have you lost interest in things? | Yes | No |
| **16** | Do you feel that you are a worthless person? | Yes | No |
| **17** | Has the thought of ending your life been on your mind? | Yes | No |
| **18** | Do you feel tired all the time? | Yes | No |
| **19** | Do you have uncomfortable feelings in your stomach? | Yes | No |
| **20** | Are you tired easily? | Yes | No |
|  | *Total number of yes answers* |  | |

1. **Health care access and perceived stigma**
2. In the last 12 months, did you go to a clinic or hospital to see a doctor or nurse? Y/ N
3. Do you attend the local clinic:

- Where you live? Y / N
- Where you work? Y / N

1. Do you always go to the same clinic? Y / N

If no, please explain……………………………………………………………………………..

1. What obstacles or barriers do you face when needing to access health care services? (choose all that apply)

| Transport problems |  |
| --- | --- |
| Unable to pay for services |  |
| Unable to take time off work during the day |  |
| No services available in the community where I live |  |
| No services available close to where I work |  |
| Have problems getting childcare |  |
| Language problems |  |
| Went to the clinic, but was turned away |  |
| Poor quality of services or care |  |
| Long waiting list to see the doctor or nurse |  |
| Other ………………………….. |  |

1. What would you consider as the *major* obstacle you faced when accessing health care services?

………………………………………………………………………………………………………

1. Do you feel that you are treated well at the clinic or hospital? Y / N
2. Do you feel that you are treated differently or discriminated against at the clinic because you work as a waste picker? Y / N

Please explain

……………………………………………………………………………………………………….

……………………………………………………………………………………………………….

1. Have you ever been admitted to a hospital? Y / N

112.1 If yes, when were you admitted and what was the diagnosis?

| **Year** | **Hospital name** | **Diagnosis** |
| --- | --- | --- |
| a. |  |  |
| b. |  |  |
| c. |  |  |
| d. |  |  |

1. **Quality of Life**
2. **Instructions for the interviewer:** This assessment asks how you feel about your quality of life, health, or other areas of your life. If you are unsure about which response to give to a question, **please choose the one** that appears most appropriate. Please keep in mind your standards, hopes, pleasures and concerns. We ask that you think about your life **in the last two weeks.**

|  | **Very poor** | **Poor** | **Neither poor nor good** | **Good** | **Very good** |
| --- | --- | --- | --- | --- | --- |
| 114.1 How would you rate yw would you rate stionnaires the diagnosis:items:)ire. I thought we could choose one that bnour quality of life? | 1 | 2 | 3 | 4 | 5 |

The following questions ask you how **good or satisfied** you have felt about various aspects of your life over the last two weeks.

|  | **Very dissatisfied** | **Dissatisfied** | **Neither satisfied nor dissatisfied** | **Satisfied** | **Very satisfied** |
| --- | --- | --- | --- | --- | --- |
| 114.2 How satisfied are you with your health? | 1 | 2 | 3 | 4 | 5 |
| 114.3 How satisfied are you with your access to health care services? | 1 | 2 | 3 | 4 | 5 |
| 114.4 How satisfied are you with your capacity to work? | 1 | 2 | 3 | 4 | 5 |

The following questions ask about **how much** you have experienced certain things in the last two weeks.

|  | **Not at all** | **A little** | **A moderate amount** | **Very much** | **An extreme amount** |
| --- | --- | --- | --- | --- | --- |
| 114.5 To what extent do you feel that physical pain prevents you from doing what you need to do? | 1 | 2 | 3 | 4 | 5 |
| 114.6 How much do you enjoy life? | 1 | 2 | 3 | 4 | 5 |
| 114.7 To what extent do you feel your life to be meaningful? | 1 | 2 | 3 | 4 | 5 |
| 114.8 How much do you fear the future? | 1 | 2 | 3 | 4 | 5 |
| 114.9 How much do you worry about your health? | 1 | 2 | 3 | 4 | 5 |

The following questions ask about **how much** you have experienced certain things in the last two weeks.

|  | **Not at all** | **A little** | **A moderate amount** | **Very much** | **Extremely** |
| --- | --- | --- | --- | --- | --- |
| 114.10 How well are you able to concentrate? | 1 | 2 | 3 | 4 | 5 |
| 114.11 How safe do you feel in your daily life? | 1 | 2 | 3 | 4 | 5 |
| 114.12 How healthy is your physical environment? | 1 | 2 | 3 | 4 | 5 |
