# Supplementary material for: Common Mental Health Disorders among Informal Waste Pickers in Johannesburg, South Africa 2018—A Cross-Sectional Study
Source: Int J Environ Res Public Health. 2019 Jul 23;16(14):2618. doi: 10.3390/ijerph16142618 (PMC6678252; doi:10.3390/ijerph16142618)
Supplement: Supplementary file 1 [file ijerph-16-02618-s001.zip › ijerph-541486-supp. Proofed/ijerph-541486- Table S1.docx]

**Table S1.** Distribution of socio-demographic features of the waste pickers in Johannesburg.

| **Socio-demographic** | **Characteristics** | **Total No.** | **Frequency (%)** |
| --- | --- | --- | --- |
| **Sex** |  | **332** |  |
|  | Males |  | 241 (72.59) |
|  | Female |  | 91 (27.41) |
|  | Missing (*n*) |  | 0 |
| **Age** |  | **332** |  |
|  | 18-30 |  | 155 (46.69) |
|  | 31-40 |  | 108 (32.53) |
|  | 41-50 |  | 40 (12.05) |
|  | 51+ |  | 29 (8.73) |
|  | Mean age (IQR) |  | 31 (IQR:27-39) |
|  | Missing (*n*) |  | 0 |
| **Education** |  | **332** |  |
|  | None |  | 13 (3.92) |
|  | Primary |  | 55 (16.57) |
|  | Secondary |  | 259 (78.01) |
|  | Tertiary |  | 5 (1.5) |
|  | Missing (*n*) |  | 0 |
| **Living adjacent to the landfill** |  | **328** |  |
|  | Yes |  | 97 (29.57)) |
|  | No |  | 231 (70.43) |
|  | Missing (*n*) |  | 4 |
| **Years working, Mean (IQR)** |  | **326** |  |
|  | 0-5 |  | 177 (54.29) |
|  | 6-10 |  | 80 (24.54) |
|  | 10+ |  | 69 (21.17) |
|  | Mean years (IQR) |  | 5 (IQR 3-10). |
|  | Missing (*n*) |  | 6 |
| **Born in South Africa** | Yes  No |  | 62 (18.67)  270 (81.33) |
|  | *Missing (n)* |  | 0 |
| ***Average_monthly income*** |  | **328** |  |
|  | *>R 1000.00* |  | 91(27.74) |
|  | R1000 –R3000 |  | 189(57.62) |
|  | R3000- R5000 |  | 37 (11.25) |
|  | R6000-10 000 |  | 8 (2.44) |
|  | R10 000.00 + |  | 3 (0.95) |
|  | Missing (n) |  | 4 |
| **Smoking** |  | **322** |  |
|  | Yes |  | 222 (68.94) |
|  | No |  | 100 (31.06) |
|  | Missing(n) |  | 10 |
| **Current_alcohol consumption** |  | **332** |  |
|  | Yes |  | 258 (77.71) |
|  | No |  | 74 (22.29) |
